# Increased intake of marine fish contributed to a decreased odds of comorbid depressive symptoms and coronary heart disease in Chinese adults

**DOI:** 10.3389/fnut.2024.1521124

**Published:** 2025-01-17

**Authors:** Yuncao Fan, Wei Chen, Wenhui Lin, Jungu Jin, Enyu Lou, Jiaying Lao, Yu-Hsin Chen, Jianzhi Shao, Qizeng Wang, Qingxi Jiang, Fan Wang, Jinzhong Xu, Yanlong Liu, Bo Yang

**Affiliations:** ^1^Department of Cardiovascular Medicine, Affiliated Wenling Hospital, Wenzhou Medical University, Wenling, China; ^2^School of Public Health, Wenzhou Medical University, Wenzhou, China; ^3^School of Mental Health, Wenzhou Medical University, Wenzhou, China; ^4^Beijing Hui-Long-Guan Hospital, Peking University, Beijing, China; ^5^Key Laboratory of Psychosomatic Medicine, Inner Mongolia Medical University, Inner Mongolia, China; ^6^Department of Clinical Pharmacy, Affiliated Wenling Hospital, Wenzhou Medical University, Wenling, China

**Keywords:** fish, depression, coronary heart disease, comorbidity, nutrition epidemiology

## Abstract

**Backgrounds:**

Increased consumption of fish has beneficial impacts upon emotional health; however, this benefit for comorbid depressive symptoms and coronary heart disease (DCHD) is not fully clear. We aimed to investigate the relationship between consumption of marine fish and DCHD in Chinese adults.

**Methods:**

A cross-sectional study was conducted in 1,106 participants aged 25–95 years living in Taizhou, China. Fish intakes were assessed by using a validated food frequency questionnaire, with their tertiles as category levels of ≤1 time/week, 2–6 times/week, and ≥7 times/week. Coronary heart disease (CHD) was diagnosed using the coronary angiography, while the concurrent depressive symptoms was indicated using ≥8 scores from hospital anxiety and depression scales (HADS). Primary measurements were the prevalent DCHD, presented as multivariate-adjusted odds ratios (ORs) with 95% confidence intervals (CIs).

**Results:**

A total of 932 participants were included, 88 (9.44%) participants with depressive symptoms, 477 (51.18%) CHD, and 106 (11.37%) DCHD, respectively. Participants at the highest tertile of fish intake have a lower odds of DCHD compared with those at the lowest (OR: 0.34, 95% CI: 0.20, 0.58), with 42% reductions in odds of DCHD for per one-tertile (3 times/week) increase (OR: 0.58, 95% CI: 0.45, 0.76). The beneficial associations were pronounced with decreased odds of depressive symptom (OR: 0.31, 95% CI: 0.20, 0.47), but not with CHD (OR: 0.87, 95% CI: 0.59, 1.29).

**Conclusions:**

Increased consumption of marine fish is associated with decreased severity of depressive symptoms, which might have great benefits toward comorbid depressive symptom and with coronary heart diseases.

## 1 Introduction

Comorbid depressive symptom and coronary heart disease (DCHD) is a combination of two diseases reported to pose significant public burdens on individuals and society (1, 2). Depression often co-occurred with coronary heart disease (CHD), with two to three times higher rate in CHD patients than the general population (3). Depression and CHD may have shared a common pathogenesis such as increased productions of pro-inflammatory cytokines, endothelial dysfunction, and hyperactivity of the hypothalamic-pituitary-adrenal (HPA) axis (4–6). However, the understanding of depression and CHD as a single comorbid phenomenon remains limited, which poses challenges for the prevention of DCHD in general population (7).

Increased consumption of fish (specifically marine fish) was found to be beneficial in preventing against depression (8) or CHD (9, 10) in the past. Results of several cohorts demonstrated fish intake was associated with reduced risk of either depression (11, 12) or CHD (13). At mechanistic levels, marine fish is a readily-accessible and well-documented dietary source of n-3 fatty acids (14, 15), which is reported to possess a remarkable anti-inflammatory capacity (16, 17), alleviate endothelial dysfunction (18), diminish the formation of atherosclerotic plaques (19), moreover modulate the synthesis, release, and function of serotonin (20). Moreover, fish is also rich in high-quality animal proteins and micronutrients (21), such as vitamin D, folate, and vitamin B12 that were reported to be beneficial in attenuating the pro-inflammatory reactions in patients with depression (22–24) or cardiometabolic disorders (25–27). Despite the abundance of studies suggesting depression and CHD share a common pathogenesis, as well as the benefits of marine fish intake toward either depression or CHD, a critical gap remains in our understanding of the differential contributions of marine fish intake to somatic vs. psycho health.

To further clarify the role of marine fish intake in preventing against mind-body disorder, we analyzed the baseline data from one recent ongoing cohort in Taizhou, China. Taizhou, an eastern coastal city in China, has been the focus of research on nutrition for over a decade (28–30), particularly in the context of marine-sourced foods [primarily large (31) and small yellow croaker (32)] due to its unique location and dietary culture. The populace of Taizhou adheres to the traditional Jiangnan Diet (33, 34), renowned for its well-balanced dietary structure, consisting of high intake of fish and vegetables and minimal oil cooking styles. The current study was aimed to further extend our understanding of the relationship between marine fish intake and DCHD. We hypothesized that increased consumption of marine fish would have a beneficial association with DCHD in this population.

## 2 Materials and methods

### 2.1 Study participants

**Figure 1:**
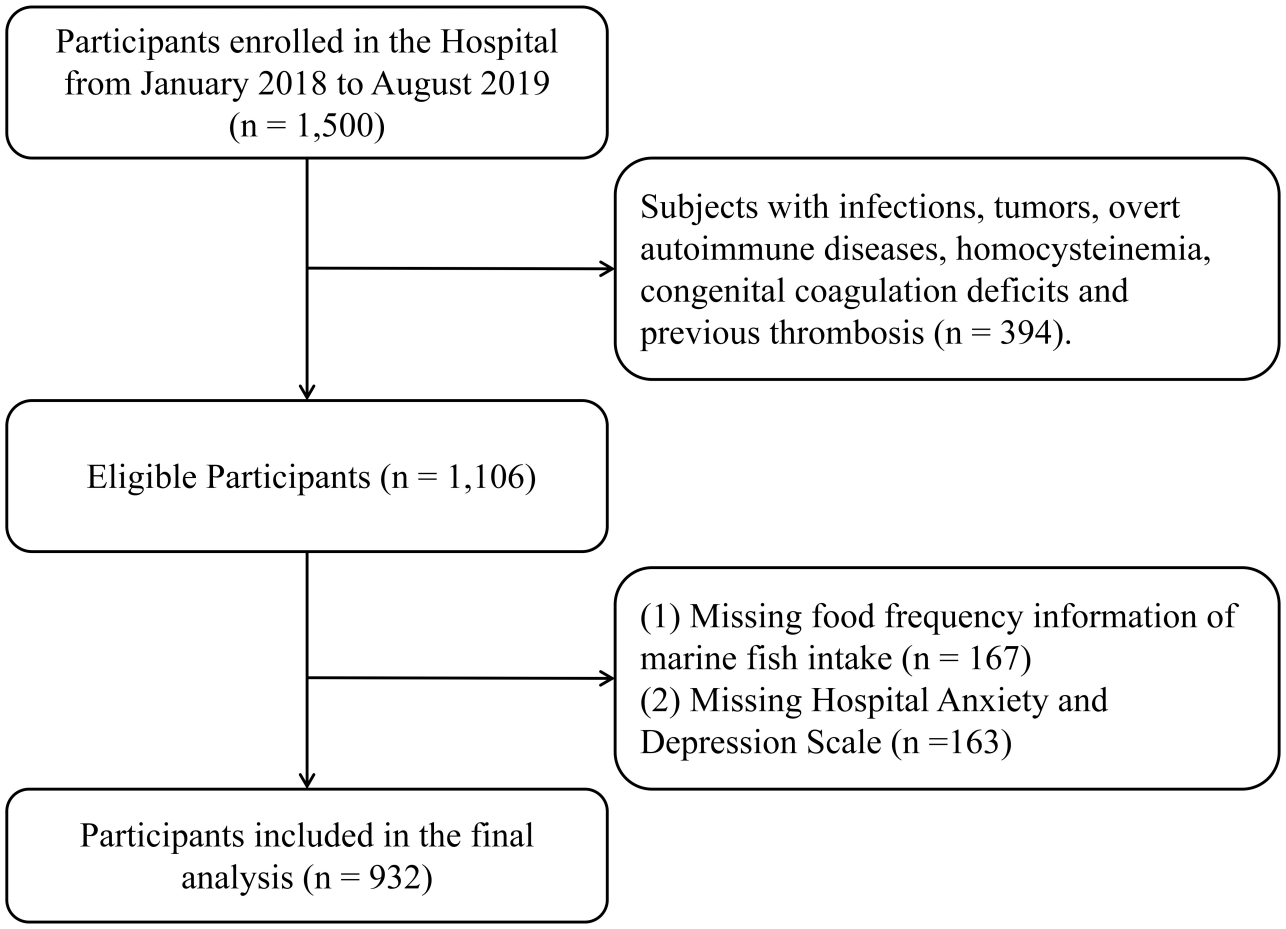
Flowchart selection of study participants.

The present cross-sectional analysis was performed using the baseline data from an ongoing cohort study in Taizhou City, Zhejiang Province, China. The original plan for the cohort was to recruit 3,000 general patients at baseline through two phases, as described in our previous studies (30, 35). In brief, the first phase aimed to recruit 1,500 persons who made appointments with and/or visited the cardiology department by posting posters at Wenling Hospital affiliated to Wenzhou Medical University, Zhejiang Province. The theoretical sample size of this study was set at 1,056 individuals to provide a specific relative precision of 1% (Type I error, 0.05; Type II error, 0.01), taking into account the prevalence of CHD in China (10.2‰) (36). The sample size was calculated based on the formula for observational cross-sectional design: $n=\frac{Z_{\alpha /2}^{2}*\left(1-p\right)*p}{\delta^{2}}$, where *Z*_α/2_ is the critical value for a two-tailed test, *p* is the estimated prevalence of events, and δ is the margin of error. In this study, after the exclusion of 394 individuals with other comorbidities (such as infection, tumors, etc.), 1,106 general patients were eligible to visit the cardiology department with clinical manifestations of suspected CHD. The final study included 932 participants due to missing FFQ data (*n* = 167) and depression scales (*n* = 163) (Figure 1). The study protocol was approved by the Institutional Research Ethics Committee of Affiliated Wenling Hospital of Wenzhou Medical University (KY-2017-2052-01). The study adheres to the Declaration of Helsinki. All participants were informed of the risks, benefits, goals and methods of the study, written consent was provided before data collection.

### 2.2 Anthropometrical measurements and questionnaire interview

All anthropometric and demographic data were measured by trained medical staff using standard protocols. Demographic data included age, gender, height, and weight. Body mass index (BMI) was calculated by dividing the participant's weight (kg) by height (meter) squared. The trained investigators obtained historical information using a standard questionnaire, including alcohol consumption, smoking, past medical history (hypertension, diabetes, stroke, and dyslipidemia), and related medication use.

The Food Frequency Questionnaire (FFQ) used in this study has been validated in several previous studies (37, 38), moreover adapted to better reflect the local dietary habits in various areas of China in the past (39, 40). Accordingly, the food items were first selected from the most frequently consumed items listed in the National Health and Dietary Survey in China (41) and then some unlisted, commonly consumed foods in the local area obtained from pilot test were added to reflect the local dietary habits of Taizhou. In the end, the food items were classified including salt, fruits, vegetables, milk, red meat, seafood (primarily yellow croaker), eggs, soy products, nuts, and sugar-sweetened beverages, and their details for reliability and validity analyses of the FFQ used in the present study were shown in Supplementary Table S1.

### 2.3 Clinical assessments

Depression status was evaluated by the hospital-based anxiety and depression scales (HADS) (42). HADS is a self-report questionnaire commonly used to assess levels of anxiety and depression in individuals. It consists of 14 items, with seven items each assessing anxiety and depression. The scale is designed to provide a quick and reliable measure of emotional distress in both clinical and non-clinical settings. Each item is scored on a Likert scale, with scores ranging from 0 to 3, and the total scores for anxiety and depression subscales can range from 0 to 21 (43, 44). HADS scores ≥8 indicate the presence of prevalent depressive symptom, while the scores ranging from 8 to 10 are diagnosed as minor depressive symptom. HADS scores ≥11 are indicative of moderate to severe depressive symptom.

Since participants recruited had clinical manifestations of suspected CHD, cardiologists were obliged to perform a thorough cardiovascular risk assessment for all study participants. All study participants underwent a coronary angiography, which was a procedure where a thin, flexible catheter was inserted into the coronary arteries. By introducing contrast agents and employing X-ray technology, cardiologists were able to visualize the interior of the arteries. During the coronary angiography, if it was observed that the degree of stenosis in any of the coronary arteries exceeded 50%, this condition was defined as indicative of coronary artery disease; subsequently referred to as coronary heart disease (CHD) subjects in this study.

### 2.4 Biochemical measurements

Anthropometrical measurements were performed by trained nurses using standard protocols. Fasting blood samples of participants were collected in the morning and were left at room temperature for 30 min, then centrifuged at 4,000 r/min for 10 min to isolate serum. Measurement of serum triglycerides (TG), total cholesterol (TC), glycated hemoglobin (HbA1C), fasting glucose (FBG), serum creatinine (Cr), serum uric acid (UA), blood urea nitrogen (BUN), Alanine aminotransferase (ALT), and Aspartate aminotransferase (AST) levels were determined by standard procedures at the affiliated Wenling hospital.

### 2.5 Statistical analysis

The normal-distribution data were expressed as the mean ± SD (standard deviation), while the skewed data were expressed as the median ± IQR (quartile range) and were log-transformed before statistical analyses. We standardized marine fish intake into an equidistant variable indicator: once time per week or less (low level), 2–6 times per week (moderate level), and ≥7 times per week (high level). *p* for trend across tertiles of marine fish intake in the continuous and categorical variables was calculated by a generalized liner model (GLM) and the chi-square test, respectively.

Multivariate-adjusted logistic regression models were used to estimate adjusted odds ratio (OR) with 95% confidence interval (CI) for the prevalent DCHD across tertiles of marine fish intake, with the lowest quartile as a reference. OR for the prevalent DCHD per one-tertile increase in fish intake was calculated by treating the ordinal tertile number as a continuous variable in the corresponding models. *p* for trend was calculated across tertiles by entering the ordinal number as a continuous variable into the corresponding models. In secondary analyses, multivariate-adjusted linear regression models were conducted to examine the relationship of marine fish intake with HADS scores and CHD risk index. CHD risk indices were defined by the arteriosclerosis index (AI) (35) and atherogenic index of plasma (AIP) (36), with the formulas for AI = (TC- HDL)/HDL and AIP = lg (TG/HDL). In both multivariable analyses, the crude model included marine fish intake only as the independent variable. Model 1 was adjusted for age, gender, overweight/obesity. Model 2 was further adjusted for lifestyle factors including current smoker, current drinker, and salt intake. Model 3 as a full model was further adjusted for clinical factors including hypertension, diabetes mellitus (DM), stroke, dyslipidemia. Explanatory variables were modeled as following: age (< 65 years, ≥65 years), overweight/obesity (< 24.0 kg/m^2^, ≥24.0 kg/m^2^), smoking (never/former, current), drinking (never/former, current), salt intake [high-salt (≥6 g/day), low-salt (< 6 g/day)], hypertension (yes, no), DM (yes, no), stroke (yes, no), and dyslipidemia (yes, no).

Furthermore, we performed subgroup analyses stratified by age (< 65 years, ≥65 years), gender, overweight/obesity (< 24.0 kg/m^2^, ≥24.0 kg/m^2^), current smoker, current drinker, hypertension, and dyslipidemia to estimate the consistency of the findings. Interaction tests were also used to determine whether ORs differed by the subgroups. Each subgroup factor, the tertiles of marine fish intake and the respective interaction terms (subgroup factor multiplied by tertiles of marine fish intake) were simultaneously included into the models to calculate a *p* valve for the interaction. In sensitivity analysis to test robustness of the major findings, we re-analyzed data to examine if the directions of the observed association of fish intake with DCHD was potentially affected by differential depressive status based on the HADS scores. Two-sided *p* < 0.05 was considered statistically significant. Data analyses were performed by STATA version 15.0 (Stata CORP, College Station, TX).

## 3 Results

### 3.1 Study characteristics

Participants' characteristics across tertiles of marine fish intake were presented in Table 1. The study included 616 males and 316 females, with an average age of 65 years; 88 (9.44%), 477 (51.18%), and 106 (11.37%) individuals had depressive symptoms, CHD, and DCHD, respectively. As compared to individuals at the lowest tertile of fish intake, those at the highest tertile were more likely to be male, young, and overweight/obese. Individuals who consume a greater amount of fish are more likely to be alcohol drinkers and smokers, and tend to have a low-salt diet.

**Table 1:** Participants' characteristics according to the frequency tertiles of fish intake.

**Value**	**Marine fish intake**			***p* for trend**
	**T1**, ***n** =* **216 (** ≤ **1 times/week)**	**T2**, ***n** =* **270** **(2–6 times/week)**	**T3**, ***n** =* **446 (**≥**7 times/week)**	
**Socio-demographics**				
Male/female, %	117/99 (54.17/45.83)	180/90 (66.67/33.33)	319/127 (71.52/28.48)	< 0.001
Age (≥65 years), %	117 (54.17)	141 (52.22)	191 (42.83)	0.003
**Lifestyle factors**				
Current smoker, %	92 (42.59)	148 (54.81)	252 (56.50)	0.002
Current drinker, %	19 (8.80)	40 (14.81)	70 (15.70)	0.024
High-salt intake (>6 g/d), %	162 (75.00)	187 (70.04)	295 (66.74)	0.031
**Clinical assessments**				
Overweight/obesity, %	102 (49.51)	126 (48.84)	249 (57.24)	0.034
Diabetes mellitus, %	50 (25.00)	52 (28.46)	74 (25.87)	0.857
Hypertension, %	132 (62.26)	153 (57.74)	283 (64.32)	0.410
Dyslipidemia, %	137 (64.32)	189 (70.79)	298 (67.27)	0.631
CHD, %	136 (62.96)	170 (62.96)	277 (62.11)	0.809
Depression, %	75 (34.72)	59 (21.85)	60 (13.45)	< 0.001
Comorbid depression and CHD, %	42 (19.44)	32 (11.85)	32 (7.17)	< 0.001
**Concurrent medications**				
Anti-hypertensive drugs, %	132 (61.11)	180 (66.67)	299 (67.04)	0.166
Hypoglycemic drugs, %	50 (23.15)	64 (23.70)	100 (22.42)	0.786
**Biochemical indicators**				
FBG (mmol/L)	5.23 (4.62, 6.08)	5.23 (4.68, 6.27)	5.26 (4.79, 6.45)	0.413
HbA1C (%)	6.20 (5.90, 6.80)	6.30 (5.90, 6.90)	6.20 (5.90, 7.00)	0.655
Cr (μmol/L)	70.60 (57.85, 84.80)	71.70 (61.75, 82.40)	70.40 (60.18, 85.30)	0.907
UA (μmol/L)	339.00 (288.75, 409.00)	352.00 (291.00, 424.00)	353.00 (294.00, 415.00)	0.093
BUN (μmol/L)	5.29 (4.43, 6.82)	5.50 (4.52, 6.55)	5.56 (4.67, 6.58)	0.309
ALT (U/L)	18.30 (13.00, 28.18)	20.80 (15.28, 28.35)	18.95 (14.35, 27.50)	0.974
AST (U/L)	21.70 (17.40, 28.65)	22.95 (18.88, 29.00)	20.85 (17.68, 26.63)	0.205
TC (mmol/L)	4.27 (3.43, 5.20)	4.24 (3.49, 4.89)	4.41 (3.64, 5.21)	0.097
TG (mmol/L)	1.45 (1.15, 2.10)	1.48 (1.11, 2.01)	1.42 (1.04, 2.13)	0.754
HDL-C (mmol/L)	1.05 (0.7, 1.20)	0.98 (0.83, 1.13)	1.02 (0.88, 1.20)	0.615
LDL-C (mmol/L)	2.91 (2.27, 3.53)	2.88 (2.28, 3.44)	2.97 (2.36, 3.59)	0.131

T, Tertiles; CHD, Coronary heart disease; FBG, Fasting blood glucose; Cr, Creatinine; UA, Uric acid; BUN, Blood urea nitrogen; ALT, Alanine transaminase; AST, Aspartate aminotransferase; TC, Total cholesterol; TG, Triglyceride; HDL-C, High density lipoprotein; LDL-C, Low density lipoprotein. *p* for trend across tertiles of fish intake in the continuous and categorical variables was calculated by a generalized liner model (GLM) and the chi-square test, respectively.

### 3.2 Comparisons of marine fish intake between participants with and without DCHD

**Figure 2:**
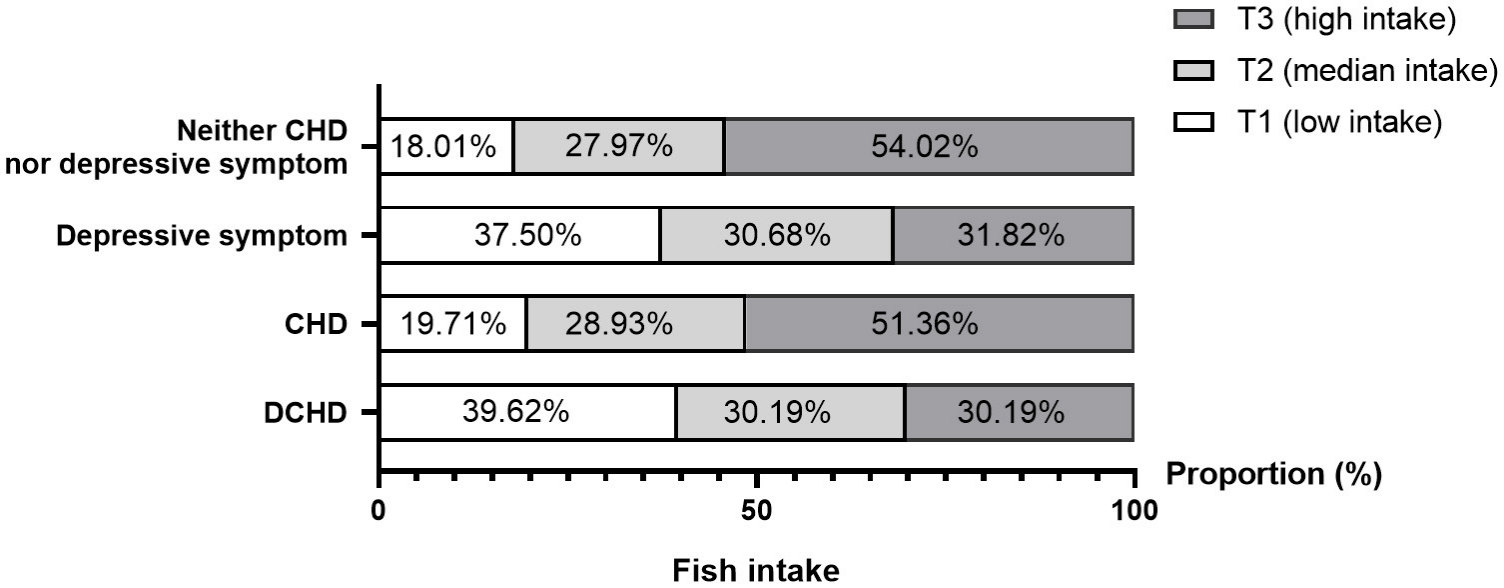
The proportions of fish intake among the participants with or without DCHD.

Figure 2 showed the comparable distributions of marine fish intake between participants with or without DCHD. The proportion of marine fish intake decreased in individuals with either depressive symptom (χ^2^ = 17.78, *p* < 0.001) or DCHD (χ^2^ = 23.74, *p* < 0.001) compared with those with neither CHD nor depressive symptom, whereas the difference was not found in the CHD participants (χ^2^ = 0.54, *p* = 0.764).

### 3.3 Marine fish intake and prevalent DCHD

Associations of marine fish intake with DCHD were shown in Table 2. Participants at the highest tertile of marine fish intake had a lower odds of DCHD compared with those at the lowest tertile (OR = 0.56, 95% CI: 0.33, 0.96), with 42% reductions in odds of DCHD for per one-tertile (3 times/week) increase (OR = 0.58, 95% CI: 0.45, 0.76). The multivariate-adjusted ORs were 0.31 (95% CI: 0.20, 0.47) for depressive symptom and 0.87 (95% CI: 0.59, 1.29) for CHD, respectively. Additionally, higher intake of marine fish was significantly associated with lower scores from the HADS assessments (β: −0.302,95% CI: −0.471, −0.133) (Supplementary Table S2), with a 0.143-score reduction in the HADS for per one-tertile increase of fish intake (β: −0.143,95% CI: −0.227, −0.059). In contrast, no significant associations were found between fish intake and either AI (β: −16.158,95% CI: −37.345, 5.028) or AIP (β: −0.071,95% CI: −0.181, 0.039) (Supplementary Table S3).

**Table 2:** Associations between marine fish and depression, CHD, or their comorbidity.

**Diseases**	**Fish**	**Cases/participants**	**Crude model**		**Model 1**		**Model 2**		**Model 3**	
			**OR (95%CI)**	***p*** **value**	**OR (95%CI)**	***p*** **value**	**OR (95%CI)**	***p*** **value**	**OR (95%CI)**	***p*** **value**
Depression	T1 (ref)	75/216								
	T2	59/270	0.53 (0.35, 0.79)	0.002	0.54 (0.35, 0.82)	0.004	0.55 (0.36, 0.83)	0.005	0.59 (0.38, 0.92)	0.019
	T3	60/446	0.29 (0.20, 0.43)	< 0.001	0.30 (0.20, 0.45)	< 0.001	0.30 (0.20, 0.46)	< 0.001	0.31 (0.20, 0.47)	< 0.001
	Per one-tertile increase		0.54 (0.45, 0.66)	< 0.001	0.55 (0.45, 0.67)	< 0.001	0.55 (0.45, 0.68)	< 0.001	0.55 (0.45, 0.69)	< 0.001
CHD	T1 (ref)	136/216								
	T2	170/270	1.00 (0.69, 1.45)	1.000	0.86 (0.57, 1.28)	0.451	0.84 (0.56, 1.26)	0.407	0.84 (0.55, 1.29)	0.425
	T3	277/446	0.96 (0.69, 1.35)	0.831	0.82 (0.57, 1.19)	0.305	0.84 (0.58, 1.26)	0.364	0.87 (0.59, 1.29)	0.482
	Per one-tertile increase		0.98 (0.83, 1.16)	0.809	0.91 (0.76, 1.09)	0.329	0.93 (0.77, 1.11)	0.096	0.94 (0.78, 1.14)	0.551
DCHD	T1 (ref)	42/216								
	T2	32/270	0.56 (0.34, 0.92)	0.022	0.56 (0.33, 0.94)	0.027	0.53 (0.32, 0.90)	0.019	0.56 (0.33, 0.96)	0.036
	T3	32/446	0.32 (0.20, 0.52)	< 0.001	0.33 (0.20, 0.55)	< 0.001	0.33 (0.20, 0.55)	< 0.001	0.34 (0.20, 0.58)	< 0.001
	Per one-tertile increase		0.57 (0.44, 0.72)	< 0.001	0.57 (0.44, 0.74)	< 0.001	0.57 (0.44, 0.74)	< 0.001	0.58 (0.45, 0.76)	< 0.001

T, tertiles; OR, odds ratio; CI, confidence interval; CHD, coronary heart disease; DCHD, comorbid depression and coronary heart disease. Model 1 was adjusted for age, gender, and overweight/obesity. Model 2 was adjusted for model 1 plus lifestyle factors (smoking, drinking, high-salt intake). Model 3 was adjusted for model 2 plus clinical factors (hypertension, diabetes, stroke, dyslipidemia).

**Figure 3:**
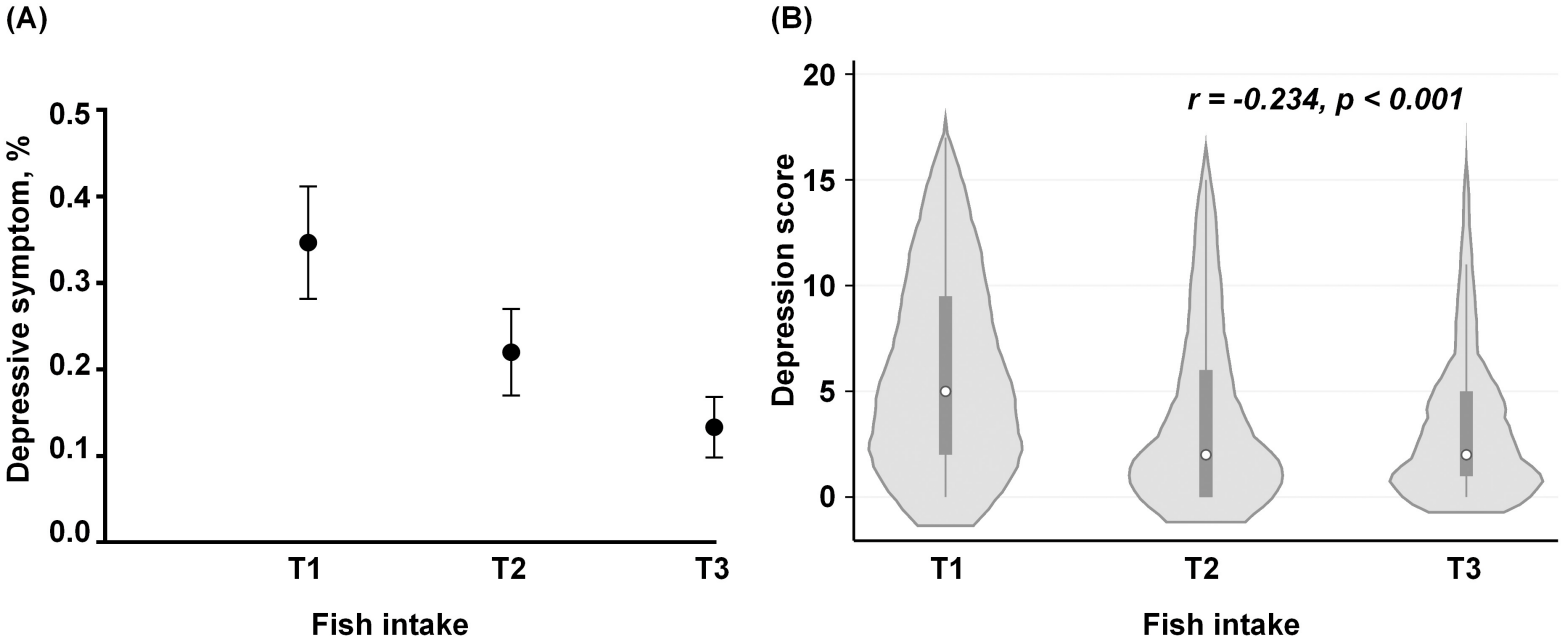
The prevalence of depression and the HADS-based scores across the tertiles of marine fish intake. **(A)** The comparisons of the proportions of prevalent depressive symptom among the tertile levels of fish intake. The black dots represent the prevalence of depression, and the ends of the vertical lines represent the 95% confidence interval (CI). **(B)** The distributions of the HADS score across the tertiles of fish intake. The white circles represent the median value of the HADS score and the small squares represent their inter-quartile range, while the vertical lines represent the 95% CI of their median values. The gray area indicates the proportion of the individuals across each level of fish intake. The frequencies of fish intake per week were standardized as an equidistant level based on their tertiles: low level (T1, ≤1 times/week), moderate level (T2, 2–6 times/week), and high level (T3, ≥7 times/week).

The comparisons of the prevalence of depressive symptom across the titles of fish intake was shown in Figure 3A. The prevalence of depressive symptom showed a significant downward trend across the tertiles of fish intake (*p* for trend < 0.001), from 34.72% (95% CI: 28.37%, 41.07%) in low-level intake to 13.45% (95% CI: 10.28%, 16.62%) in high-level intakes. Moreover, the trends in HADS-based scores across the tertiles of fish intake were shown in Figure 3B. The depression scores from HADS demonstrated a significant decrease from 5.97 (4.63) in the lowest-intake level to 3.40 (3.61) in the highest-intake levels, and a significant negative correlation was found between fish intake and depression score (r = −0.234, *p* < 0.001) (Figure 3B).

### 3.4 Subgroup analyses

Results of additional analyses stratified by age, gender, obesity status, life style factors and metabolic comorbidities were shown in Supplementary Figure S1. Higher intake of fish was associated with a higher odds of DCHD in the young and middle-aged, non-obesity participants, and those with concurrent hypertension or dyslipidemia, but not in the elderly, obesity participants, and those without current cardiometabolic diseases. No significant interaction was found between fish intake and the pre-defined subgroups on the prevalent DCHD.

### 3.5 Sensitive analysis

**Figure 4:**
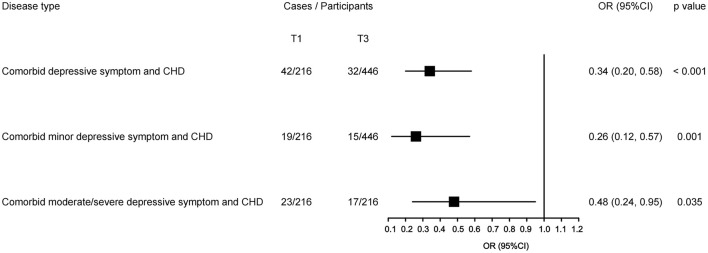
Sensitive analyses for the highest vs. lowest tertile of fish intake with comorbid different depression status with CHD. The black square presents the adjusted odds ratios (ORs) and the small bar line was their corresponding 95% confidence intervals (CIs), with the lowest tertile as a reference. The odds ratios are adjusted for the potential confounders, and *p*-values indicate a statistical significance.

When stratified by status of depressive symptoms (Figure 4), fish intakes' beneficial associations remained statistically significant with comorbid either minor depressive symptom or moderate/severe depressive symptom and CHD, and their OR values were 0.26 (95% CI: 0.12, 0.57; *p* = 0.001) and 0.48 (95% CI: 0.24, 0.95; *p* = 0.035), respectively.

## 4 Discussion

To the best of our knowledge, this current study is the first cross-sectional analysis of the relationship between marine fish intake and comorbid depressive symptom and CHD (DCHD) in China. A major finding of the present study indicated that highest marine fish intake was associated with the lowest odds of DCHD, especially with depressive symptom. Such findings extend the previous understanding of emotion-health benefits from fish intake and further provided new insights into driving the development of preventive strategies in targeting DCHD.

Compared to previous epidemiology studies, we discovered a significant negative association between marine fish intake and the prevalence of DCHD, particularly depressive symptom in coastal areas of China. Moreover, the findings of subgroup analyses revealed that the benefits of marine fish consumption on DCHD cannot be potentially changed by age, gender, and disease status. Nevertheless, limited data may have delved into the potential impacts of fish consumption in mitigating the development of DCHD. In support of the current findings, a recent meta-analysis of 10 cohorts revealed per one-serving/week increment of fish intake resulted in 11% reductions in the risk of depression (12). In contrast, a cross-sectional study conducted in Dutch older subjects with a history of myocardial infarction found that fish intake was not related with either depressive symptoms or dispositional optimism (45). Moreover, a large national survey found higher consumption of breaded fish to be associated with increased risk of greater depressive symptom, while total fish consumption, non-breaded fish and shellfish were not (46). There are several possible reasons to explain the discrepancy between the above-mentioned findings. On the one hand, the elderly participants may have potential clinical comorbidities such as hypertension, thereby probably reducing the observed benefit in the present study. On the other hand, the fish type and cooking style are important confounding factors that may attenuate the beneficial impact of fish intake on depression (46, 47).

We found a null association between marine fish consumption and the prevalent CHD, which diverged from the established conclusions in most of the previous studies. Prospective cohort-based evidence suggested high fish intake contributed to a lower cardiovascular risk (48–50), and this benefit was also supported in a recent meta-analysis based on 38 cohorts that concluded a negative association between fish intake and both CHD incidence and mortality (51). Several possible reasons may have explained the observed discrepancy between the above-mentioned studies and our results. Most of the patients who were enrolled in our study were at high coronary risks or general patients with an average age of 65 years, therefore their cardiovascular benefit attributable to fish intake may have been at least or partially diminished. Moreover, it is worth noting that majority of the patients were undergoing the treatment of lipid-lowering drugs, which may potentially overshadow the benefits of fish consumption in preventing against CHD. Third, our participants were recruited from hospital outpatient clinics, rather than from the general community, which may have a certain level of selection bias. The current findings need to be interpreted with more cautions; therefore, a prospective study will be conducted to test this observation in the general populations.

Several potential mechanisms may have explained the beneficial role of marine fish intake in preventing against DCHD. First, marine fish is a rich source of n-3 PUFAs, especially eicosatetraenoic acid (EPA) and docosahexaenoic acid (DHA) (52). One randomized controlled trial (RCTs) proved that n-3 PUFAs supplements were effective in the treatment of comorbid depression and heart failure (53). Another RCT found supplements of n-3 PUFAs to be more effective in improving depressive symptoms in CHD patients with evidence of oxidative stress than in general CHD patients (54). Several mechanisms have been proposed to explain the beneficial effects of LC n-3 PUFAs on depression and CHD (55). Key guidelines from the International Society for Nutritional Psychiatry indicate that the clinical use of n-3 PUFAs in major depressive disorder with high levels of inflammatory markers or low n-3 index may be considered as an area that deserve future research (56). In addition, n-3 PUFAs contribute to CHD prevention through various mechanisms, including improving endothelial function, reducing lipid accumulation, lowering vascular inflammation, inhibiting plaque formation, and enhancing plaque stability (57). Third, vitamins D and minerals from fish may have a protective effect on depression (58). Vitamin D3 influences melatonin production and circadian rhythms directly through its role in the synthesis pathways involved, potentially improving symptoms of depression by modulating these biological rhythms. Additionally, vitamin D3 impacts various hormones and neurotransmitters, indirectly affecting circadian rhythms and contributing to improved mood and reduced symptoms of Seasonal Affective Disorder (SAD) (59, 60). Fourthly, fish is valued for its high-quality protein, a composition rich in essential amino acids similar to human protein, easily digestible and absorbable due to its short and thin muscle fibers (61). Maintaining an adequate protein intake assumes critical significance in the prevention of DCHD, as it represents an essential nutrient crucial for human physiological functioning.

## 5 Limitation

Several potential limitations should be considered in interpreting the present results. First, the study was a cross-sectional analysis, limiting the ability to establish a causal inference. Longitudinal studies with larger sample sizes are required to confirm the present findings. Second, fish intake was assessed using the FFQ, which is subject to recall bias and thus may not accurately reflect their real intakes. Further measurements of blood n-3 PUFAs would be performed to verify the observed benefits for marine fish intake. Third, this study did not differentiate between different types of fish (e.g., oily fish). Of note, we assessed the participants' habitual intake of marine fish intake using a face-to-face FFQ and the prevalent dietary preference for large yellow croakers were well-known in local persons lived in Taizhou, China. Fourth, depressive symptom was diagnosed using the HADS report, which may have led to misclassification bias. Therefore, the marine fish intakes' benefits for depression need to be explained with more cautions. Fifth, although all of results were adjusted for various potential confounders, we cannot rule out the presence of some unknown or unmeasured factors that may partially affected the association estimations. Finally, the study was conducted in the limited number of Taizhou population in China, limiting the generalizability of the findings to the whole Chinese.

## 6 Conclusion

In conclusion, marine fish intake was beneficially associated with DCHD, especially with depression, which may extend the previous knowledge of fish intake in promoting the public psychosomatic health of general population.

## Data Availability

The original contributions presented in the study are included in the article/Supplementary material, further inquiries can be directed to the corresponding authors.
