# Supplementary material for: Increased intake of marine fish contributed to a decreased odds of comorbid depressive symptoms and coronary heart disease in Chinese adults
Source: Front Nutr. 2025 Jan 17;11:1521124. doi: 10.3389/fnut.2024.1521124 (PMC11782035; doi:10.3389/fnut.2024.1521124)
Supplement: Supplementary file 1 [file Table_1.docx]

Supplementary Material

**Table S1.** Reliability and validity analyses of the Food Frequency Questionnaire.

| Test | Estimate | Value |
| --- | --- | --- |
| Reliability analysis |  |  |
| Evaluation of the internal consistency | Cronbach's alpha coefficient | 0.545 |
| Construct validity test |  |  |
| Kaiser-Meyer-Olkin Measure of Sampling Adequacy | KMO | 0.653 |
| Bartlett test of sphericity | Chi-square | 628.73 |
|  | Degrees of freedom | 45 |
|  | p-value | <0.001 |


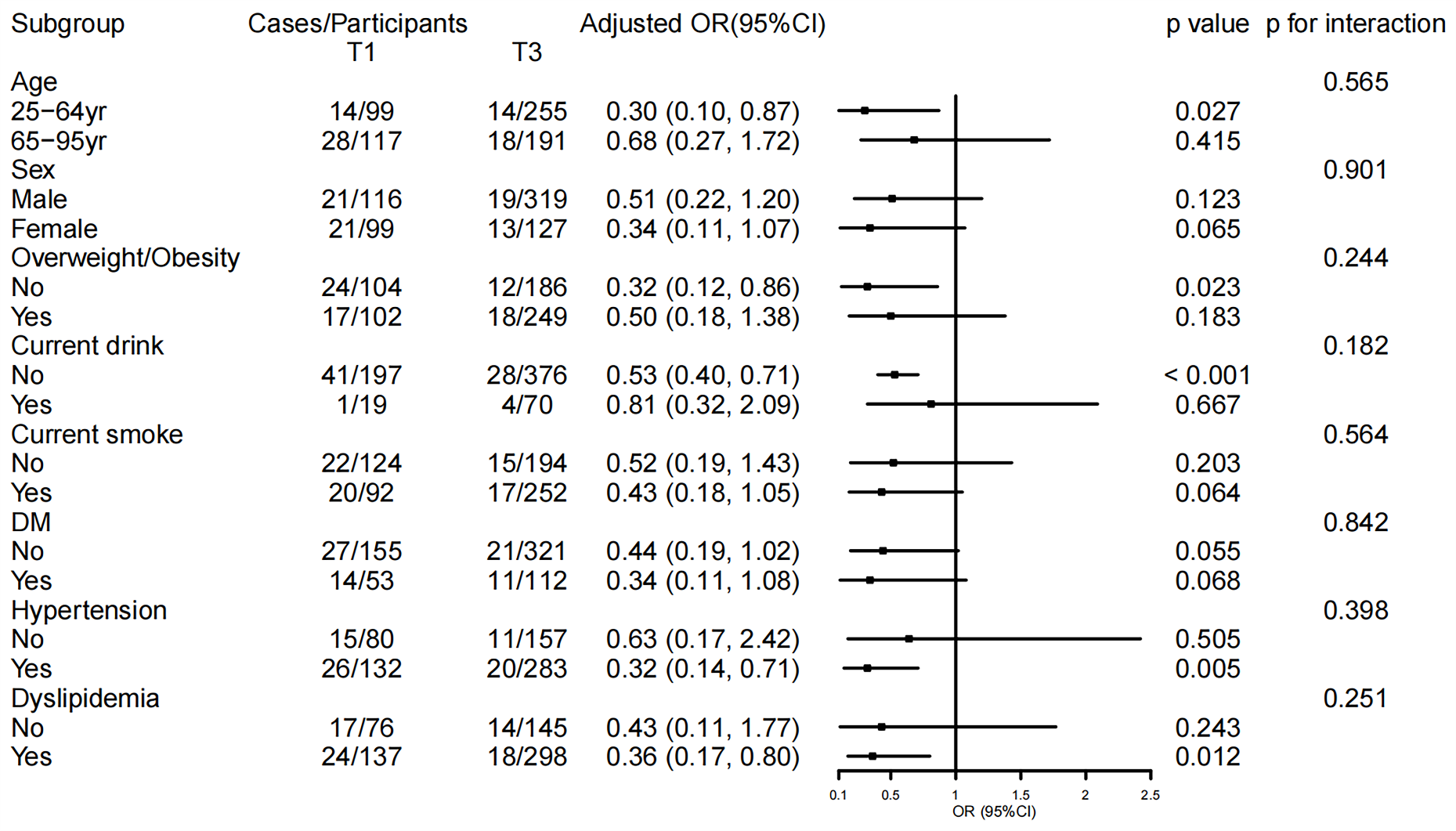


**Figure S1.** Subgroups analyses for the highest vs. lowest tertile of marine fish intake in relation to DCHD.

**Table S2.** Linear regression of HADS score associated with marine fish intake.

| HADs score | Fish intake | Crude model |  | Model 1 |  | Model 2 |  | | Model 3 |  | |
| --- | --- | --- | --- | --- | --- | --- | --- | --- | --- | --- | --- |
|  |  | β (95%CI) | *p* value | β (95%CI) | *p* value | β (95%CI) | | *p* value | β (95%CI) | | *p* value |
|  | T1 (ref) |  |  |  |  |  | |  |  | |  |
|  | T2 | -0.317 (-0.480, -0.154) | <0.001 | -0.314 (-0.480, -0.148) | <0.001 | -0.297 (-0.465, -0.129) | | 0.001 | -0.267 (-0.445, -0.090) | | 0.003 |
|  | T3 | -0.458 (-0.605, -0.313) | <0.001 | -0.438 (-0.588, -0.289) | <0.001 | -0.335 (-0.495, -0.174) | | <0.001 | -0.302 (-0.471, -0.133) | | <0.001 |
|  | per one-tertile increase | -0.222 (-0.294, -0.150) | <0.001 | -0.211 (-0.285, -0.138) | <0.001 | -0.159 (-0.238, -0.079) | | <0.001 | -0.143 (-0.227, -0.059) | | 0.001 |

Model 1 was adjusted for age, gender, overweight/obesity.

Model 2: model 1 plus adjustment for lifestyle factors (smoking, drinking, salt intake).

Model 3: model 2 plus adjustment for clinical factors (CHD, hypertension, diabetes, stroke, dyslipidemia).

**Table S3.** Linear regression of high CHD risk index associated with marine fish intake.

| High CHD risk index | Fish intake | Crude model |  | Model 1 |  | Model 2 |  | Model 3 |  |
| --- | --- | --- | --- | --- | --- | --- | --- | --- | --- |
|  |  | β (95%CI) | *p* value | β (95%CI) | *p* value | β (95%CI) | *p* value | β (95%CI) | *p* value |
| AI |  |  |  |  |  |  |  |  |  |
|  | T1 (ref) |  |  |  |  |  |  |  |  |
|  | T2 | -16.640 (-37.927, 4.647) | 0.125 | -18.302 (-40.590, 3.986) | 0.107 | -18.008 (-40.502, 4.486) | 0.116 | -18.291 (-41.490, 4.908) | 0.122 |
|  | T3 | -16.118 (-35.438, 3.201) | 0.102 | -18.276 (-38.669, 2.117) | 0.079 | -17.577 (-38.160, 3.005) | 0.094 | -16.158 (-37.345, 5.028) | 0.135 |
|  | per one-tertile increase | -7.106 (-16.585, 2.364) | 0.141 | -8.097 (-18.088, 1.895) | 0.112 | -7.758 (-17.849, 2.333) | 0.132 | -6.946 (-17.335, 3.444) | 0.190 |
| AIP |  |  |  |  |  |  |  |  |  |
|  | T1 (ref) |  |  |  |  |  |  |  |  |
|  | T2 | 0.019 (-0.090, 0.128) | 0.727 | 0.004 (-0.103, 0.111) | 0.939 | -0.001 (-0.110, 0.108) | 0.983 | 0.005 (-0.109, 0.120) | 0.928 |
|  | T3 | -0.019 (-0.118, 0.080) | 0.703 | -0.070 (-0.168, 0.028) | 0.160 | -0.067 (-0.171, 0.038) | 0.213 | -0.071 (-0.181, 0.039) | 0.207 |
|  | per one-tertile increase | -0.013 (-0.061, 0.036) | 0.603 | -0.040 (-0.087, 0.008) | 0.105 | -0.036 (-0.088, 0.015) | 0.166 | -0.040 (-0.094, 0.015) | 0.152 |

Model 1 was adjusted for age, gender, overweight/obesity.

Model 2: model 1 plus adjustment for lifestyle factors (smoking, drinking, salt intake).

Model 3: model 2 plus adjustment for clinical factors (depressive symptom, hypertension, diabetes, stroke, dyslipidemia).
